# CD200 and CD200R Expression on Peripheral Blood Lymphocytes and Serum CD200 Concentration as a New Marker of Endometriosis

**DOI:** 10.3390/jcm9093035

**Published:** 2020-09-21

**Authors:** Monika Abramiuk, Ewelina Grywalska, Izabela Korona-Głowniak, Paulina Niedźwiedzka-Rystwej, Grzegorz Polak, Jan Kotarski, Jacek Roliński

**Affiliations:** 1 1st Department of Gynecological Oncology and Gynecology, Medical University of Lublin, 20-081 Lublin, Poland; grzegorz.polak@umlub.pl (G.P.); jan.kotarski.gabinet@gmail.com (J.K.); 2 Department of Clinical Immunology and Immunotherapy, Medical University of Lublin, 20-093 Lublin, Poland; jacek.rolinski@gmail.com; 3 Department of Pharmaceutical Microbiology, Medical University of Lublin, Chodzki 1, 20-093 Lublin, Poland; iza.glowniak@umlub.pl; 4 Institute of Biology, University of Szczecin, 71-412 Szczecin, Poland; paulina.niedzwiedzka-rystwej@usz.edu.pl

**Keywords:** endometriosis, CD200, CD200R, negative co-stimulation, non-invasive markers

## Abstract

The causes of endometriosis (EMS) remain unknown; however, a number of immunological abnormalities contribute to the pathogenesis of the disease. The cluster of differentiation-200 (CD200) and its receptor (CD200R) maintain peripheral self-tolerance by negatively regulating immune responses. In this comparative cross-sectional study, we investigated the expression of CD200 and CD200R on T and B lymphocytes and the serum level of soluble CD200 (sCD200) using flow cytometry and ELISA, respectively. Peripheral blood samples were collected from 54 female patients and 20 healthy, age-matched controls. Results were tested for correlation with disease severity and selected clinical parameters. We demonstrated that the differences in sCD200 levels (*p* = 0.001), the frequencies of CD200-positive T and B lymphocytes (*p* < 0.001 and *p* = 0.004, respectively), and the frequencies of CD200R-positive T and B lymphocytes (*p* < 0.001 for all comparisons) in the study group correlated positively with disease severity. Receiver operating characteristic (ROC) analysis indicated that aberrant expression of CD200/CD200R might serve as a marker to distinguish between EMS cases. Finally, negative co-stimulatory factors may contribute to the induction and persistence of inflammation associated with EMS. It seems that it is essential to determine whether alteration in the CD200/CD200R pathway can be therapeutically targeted in EMS.

## 1. Introduction

Endometriosis (EMS) is a benign but chronic gynecological disorder characterized by the presence of active endometrial glands and stroma outside the uterine cavity. According to different authors, the prevalence of EMS may vary from 1.5 to 15% in women of reproductive age because of methodological differences in sample collection in different populations [1,2,3].

The location of the changes is diverse; it may include the pelvic peritoneum, the pouch of Douglas, the muscular layer of the uterus, the ovaries, and the retrovaginal septum, as well as more distant locations, including the diaphragm. Lesions may deeply infiltrate local tissues or may have a superficial presentation. The inconsistent demonstration of the disease is associated with a wide range of accompanying symptoms that affect the quality of life and psychological prosperity of the affected women [4,5]. The symptoms are mainly divided into two groups: Those associated with pain and those related to sub- or infertility [3,6]. The available therapeutic methods are based on symptomatic treatment, which does not modify the course of the disease. There is no effective and easy method to confirm the disease. The gold standard in its diagnosis is to perform a laparoscopy with the consequent histopathological examination [1].

Classical theories regarding the development of EMS include Dmowski’s theory. This theory assumes that the survival and development of endometrial tissue that migrates outside the uterine cavity is possible because of the local immune tolerance in its environment [7]. More recent studies consider the chronic inflammatory response as an important part of the pathomechanism of this disease. Initially, it may appear as an increased influx of cells, followed by an acute inflammatory process that involves the local vasculature, a specific cytokine profile, and the recruitment of immunocytes [8,9,10,11].

Several impairments of immune clearance were found in the microenvironment of endometrial implants. T regulatory cells (Tregs) are an important population involved in the implantation and the survival of ectopic lesions. They decrease the ability of other immunocompetent cells to effectively recognize and target endometrial antigens during menstruation [12]. Some studies have demonstrated a diminished cytotoxicity of natural killer (NK) cells in the peritoneal fluid (PF) of patients with EMS [13,14,15]. Also, altered counts of mature and immature dendritic cells (DCs), which contribute to their diminished activity, seem to be an important issue [16]. Another mechanism involves the upregulation of deposits of indoleamine-pyrrole 2,3-dioxygenase (IDO), which modulates macrophage differentiation into the tolerogenic phenotype. Its overexpression may induce the depletion of local tryptophan and the production of kynurenines—tryptophan metabolites that are powerful inducers of apoptosis in cluster of differentiation 4-positive (CD4+) T cells [17,18]. IDO expression promotes the immunosuppressive profile of DCs and stimulates the anergic status of effector T cells [18].

The cluster of differentiation-200 (CD200) is a 41–47 kilodaltons (KDa) cell surface protein with two extracellular immunoglobulin superfamily (Ig SF) domains, a transmembrane region, and a small cytoplasmic tail [19,20,21]. Primarily expressed in myeloid and endothelial cells, it is also found on thymocytes, lymphocytes, syncytiotrophoblasts, and as a soluble form (sCD200) [22]. CD200 is an important inhibitory ligand that exerts its function by binding to the CD200 receptor (CD200R) expressed on a wide range of immune cells. CD200R mediates the inhibitory roles of CD200 through its N-terminal immunoglobulin (Ig) V-type domain, initiating an intracellular-signaling cascade for suppressive immune responses [23,24].

Signals associated with the CD200/CD200R interaction have been investigated in several inflammatory diseases. It was assumed that this signaling pathway is one of the main inducers of the Treg phenotype and that it leads to the overproduction of the IDO enzyme [25]. The direct suppression of NK cells as a result of the CD200/CD200R axis interaction is an important issue [26]. It sheds new light on the source of immunosuppressive mechanisms in such a mysterious disease as EMS.

The nature of EMS is complex and the number of publications focusing on the CD200/C200R-signaling pathway is sparse. Thus, its role in EMS development remains poorly understood. The present study aimed to investigate CD200 and CD200R expression on blood lymphocytes and in the serum of EMS patients compared with healthy controls and its relation to the severity of the disease.

## 2. Materials and Methods

### 2.1. Patients and Controls

Participants included female patients from the 1st Department of Oncological Gynecology and Gynecology of the Medical University of Lublin, hospitalized due to planned laparoscopic treatment. Blood samples were taken a day before the surgical procedure. The study group consisted of 54 previously untreated women (aged 18–55) with suspicion of EMS. EMS was confirmed based on postoperative histopathological examination. The stage of the disease was assessed according to the revised American Society of Reproductive Medicine (rASRM) classification [27]. The control group consisted of 20 age-matched volunteers without suspicion of EMS. Ultimately, EMS was excluded in these women after histopathological examination. The presence of myoma or adenomyosis in participants of the study or control group resulted in exclusion from the study. Neither participants in the study group nor in the control group were taking medications affecting the immune system or hormonal treatment. Patients also did not take painkillers 24 h before blood collection. The remaining exclusion criteria included any signs of infection occurring in the four weeks prior to the enrollment, past blood transfusion, an autoimmune disease, pregnancy, lactation, oncological history, allergy, and any known immune impairment. Neither the patients from the study group nor the control group were suffering from any autoimmune diseases. The study protocol was approved by the Institutional Review Board of the Medical University of Lublin (No. KE-0254/302/2014). Written, informed consent was obtained from all participants before the beginning of the study. This study was conducted in accordance with the Helsinki Declaration.

### 2.2. Material Collection

A total of 15 mL of peripheral blood (PB) was drawn from the basilic vein of EMS patients and controls on the day before surgery after overnight fasting. To obtain serum for the measurement of sCD200 concentration, 5 mL of PB was collected using tubes containing a clotting activator. Another 10 mL was collected into ethylenediaminetetraacetic acid (EDTA)-coated tubes (EDTA; Sarstedt, Germany) for the isolation of PB mononuclear cells (PBMCs) and flow cytometry analysis. Laboratory procedures were carried out within two hours of collection.

### 2.3. Immunophenotyping

The PB samples were diluted with 0.9% magnesium (Mg^2+^)/calcium (Ca^2+^)—free phosphate-buffered saline (PBS) (Biochrome AG, Berlin, Germany) at a 1:1 ratio. The diluted samples were separated by density-gradient centrifugation by layering on 3 mL of Gradisol L (Aqua Medica, Poland; specific gravity 1.077 g/mL) and centrifuging at 700× *g* for 20 min. The PBMCs were collected with Pasteur pipettes and washed twice with Ca^2+^/Mg^2+^-free PBS for 5 min. Afterwards, the cells were suspended in 1 mL of PBS and counted in a Neubauer chamber. Their viability was assessed using trypan blue (0.4% Trypan Blue Solution, Sigma Aldrich, St. Louis, MO, USA).

**Figure A1:**
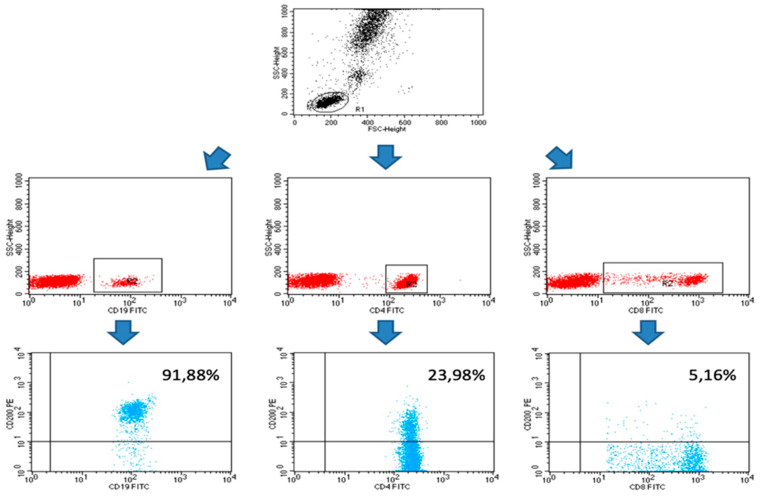
Sample analysis of CD4+/CD8+/CD200 T lymphocytes and CD19+/CD200 B lymphocytes in EMS patients.

**Figure A2:**
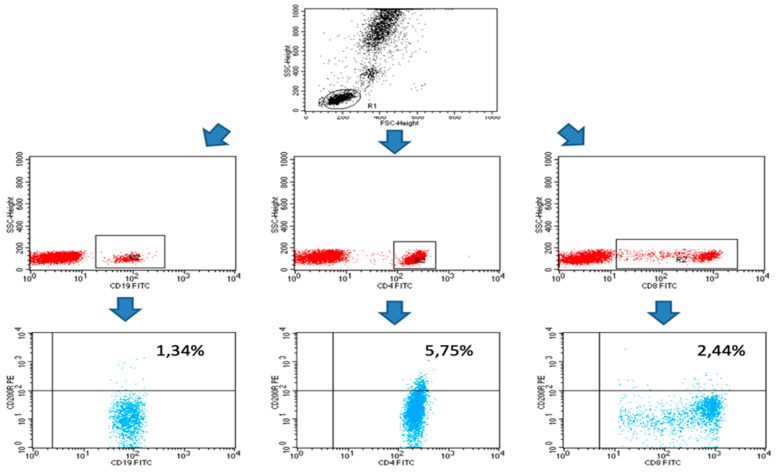
Sample analysis of CD4+/CD8+/CD200R T lymphocytes and CD19+/CD200R B lymphocytes in EMS patients.

Flow cytometry was used to assess the percentages of CD200+ and CD200R+ cells within the CD4+ T, CD8+ T, and CD19+ B lymphocyte populations. After PBMCs were isolated using the method described above, the cell suspension was divided into single tubes with 1 × 10^6^ cells per sample and incubated with the appropriate monoclonal antibodies (mAbs). We used fluorochrome-conjugated mAbs against the following markers: CD45- fluorescein isothiocyanate (FITC)/CD14- phycoerythrin (PE), mouse anti-human CD3-CyChrome, mouse anti-human CD19-FITC, mouse anti-human CD4-FITC, mouse anti-human CD8-FITC, mouse anti-human CD200-PE, and mouse anti-human CD200R-PE (BD Biosciences, San Jose, CA, USA). We also used the Human Treg Flow kit (FOXP3 Alexa Fluor 488/CD4 PE-Cyanine-5 (Cy5)/CD25 PE; BioLegend, San Diego, CA, USA) to identify the CD4+CD25+high forkhead box P3 (FOXP3+) Treg subpopulation. During analysis, the CD3+CD16+CD56+ natural killer T-like (NKT-like) cell population and CD16+CD56+ NK+ cells were also measured with anti-CD3-FITC, CD16CD56-PE, and CD45- peridinin-chlorophyll-protein (PerCP) mAbs (BD Biosciences, USA). The cells were incubated for 20 min at room temperature with 20 μL of each mAb per sample. Next, the suspension was washed twice with PBS (700× *g*, 5 min) and analyzed in a FACSCalibur flow cytometer (Becton-Dickinson, Franklin Lakes, NJ, USA) equipped with a 488-nm argon laser. Data acquisition was performed with the FACS Diva Software 6.1.3 (Becton Dickinson, Franklin Lakes, NJ, USA), including 20,000 cells per run. Analyzed data were collected using CellQuest Pro Software (Becton Dickinson, Franklin Lakes, NJ, USA). The labeled cells were examined based on lymphocyte gates at combined CD45/CD14 coordinates. The samples were gated on forward scatter vs. side scatter. The results of the flow cytometry analysis are presented as percentage of stained cells. A sample analysis for patients with EMS is shown in Appendix A, Figure A1 and Figure A2. Background fluorescence was determined using isotype-matched directly conjugated FITC-Immunoglobulin G1 (IgG1) and PE-IgG1 controls to exclude contamination and cell aggregates.

### 2.4. Measurement of sCD200 Concentration

The concentration of sCD200 was measured using the Human CD200 ELISA Kit (sensitivity 20 pg/mL; ThermoFisher Scientific, Waltham, MA, USA) according to the manufacturer’s recommendations. The results were measured with an automatic reader VICTOR3 (Perkin Elmer, Boston, MA, USA), which measures the absorbance of light in the tested material and compares it with control samples of known concentration. The WorkOut Software plotted linear curves and, based on these, the concentration of soluble antigen in the samples was calculated.

### 2.5. Measurement of Cancer Antigen 125 (CA-125) and Human Epididymis Protein 4 (HE-4) Level

Levels of the cancer antigen 125 (CA-125) and the human epididymis protein 4 (HE-4) were analyzed in preoperative samples in a central laboratory (Central Laboratory of Independent Public Teaching Hospital No. 1 in Lublin). Samples were centrifuged immediately after collection. Plasma concentrations of CA-125 and HE-4 were determined by electrochemiluminescence immunoassay (ECLIA) using the ROCHE Cobas E601 system (Roche Diagnostics GmbH, Mannheim, Germany) and the CA125 II kit or Elecsys HE4 kit (Roche Laboratories), respectively. CA-125 reference values were less than 35 U/mL. The cutoff value for the HE-4 was less than or equal to 70 pmol/L (for non-menopausal women, according to manufacturer instructions).

### 2.6. Statistical Analysis

Results from measurable parameters are presented as the mean, median, minimum, and maximum values and standard deviation. Nonmeasurable parameters are presented as means of count and percentage. The normal distribution of variables was checked using the Shapiro–Wilk test. The Student t test was used to compare independent variables and the Mann–Whitney U test was used for intergroup comparisons. Differences between more than two groups were analyzed with the Kruskal–Wallis test, ANOVA, and multiple comparisons of mean ranks (as post hoc analysis) with the Bonferroni correction. The associations between pairs of variables were assessed with Spearman’s rank correlation. Receiver operating characteristic (ROC) curves were generated for significant predictor variables of EMS. The DeLong test was used to compare the respective areas under the curve (AUC). Statistical significance was considered at *p* < 0.05. Logistic regression models were fitted to identify risk factors associated with EMS. From these models, adjusted odds ratios (OR) and 95% confidence intervals were derived; corresponding p-values were those from Wald’s test. Goodness of fit was checked using Hosmer and Lemeshow’s test. The statistical analysis was carried out using Statistica 13.3 software (StatSoft, Kraków, Poland).

## 3. Results

### 3.1. Patients and Controls

This study included 54 previously untreated women (mean age: 35.00 ± 6.20 years) with newly diagnosed endometriosis and 20 healthy individuals in the control group (mean age: 36.65 ± 6.87 years). The patients’ mean body mass index (BMI) as within the normal range and it did not differ significantly from that of the control group. The characteristics of the study and the control groups are presented in Table 1. The following types of EMS were observed in the study group: Ovarian, peritoneal, and deep infiltrating. Peritoneal adhesions were found during the surgery and described in the postoperative protocol. Infertility and dysmenorrhea were diagnosed during an interview and medical examination. There were no differences in CA-125 and HE-4 levels and in the hormonal panel between the study and control groups.

### 3.2. Frequencies of White Blood Cells and Basic Lymphocyte Subsets in EMS Patients and Controls

The differences in leukocyte count, neutrophils, and monocytes between EMS patients and controls were statistically significant (*p* = 0.015, *p* = 0.015, and *p* = 0.054, respectively; Table 2). Interestingly, EMS patients had lower frequencies of NK cells (*p* < 0.001; Table 2). Regarding the frequencies of basic lymphocyte subsets, the percentage of CD3+ T cells and the T CD3+/CD4+:T CD3+/CD8+lymphocyte ratio were significantly higher in patients than in controls (*p* = 0.021 and *p* = 0.016, respectively; Table 2). However, patients had significantly lower percentages of CD19+ B cell (*p* < 0.005).

### 3.3. Frequencies of CD200 and CD200R Expression on T and B Lymphocyte Subsets and Concentration of sCD200 in EMS Patients and Controls

Significantly higher percentages of CD4+/CD200+ T lymphocytes, CD8+/CD200+ T lymphocytes, and CD19+/CD200+ B lymphocytes were observed in EMS patients compared with the controls (*p* < 0.001, *p* < 0.001, and *p* = 0.04, respectively; Table 3). Furthermore, EMS patients had significantly lower percentages of all lymphocyte subpopulations expressing CD200R compared with controls (*p* < 0.001 for all comparisons; Table 3). The concentration of sCD200 in EMS patients was significantly lower than in the control group (*p* = 0.001; Table 3).

### 3.4. Frequencies of CD200 and CD200R on T and B Lymphocyte Subsets in Patients with Different Types of EMS

The frequencies of CD200 and its ligand on T and B lymphocytes, as well as the serum concentration of sCD200, are presented in Table 4. Significantly lower percentages of CD19+/CD200+ B lymphocytes and CD19+/CD200R+ B lymphocytes were found in patients with endometriomas (*p* = 0.049 and *p* = 0.05, respectively). The sCD200 serum level was significantly higher in these patients (*p* = 0.021) than in patients with other types of EMS. Patients with deep infiltrating endometriosis had a higher expression of CD19+/CD200+ B lymphocytes and CD8+/CD200+ T lymphocytes (*p* = 0.00034 and *p* = 0.00027, respectively). Patients with peritoneal EMS had a considerably higher percentage of the B lymphocyte subpopulation expressing CD200 (*p* = 0.029).

### 3.5. Frequencies of CD200 and CD200R on T and B Lymphocyte Subsets in Patients with EMS and Adhesions, Infertility, or Dysmenorrhea

Table 5 presents the frequencies of CD200 and CD200R on T and B lymphocyte subsets, as well as the serum concentration of sCD200. Significantly higher percentages of CD19+/CD200+ B lymphocytes, CD8+/CD200+ T lymphocytes, and CD8+/CD200R+ T lymphocytes were found in patients with EMS and concomitant adhesions (*p* = 0.024, *p* = 0.0056, and *p* = 0.024, respectively) compared with those with EMS but without peritoneal adhesions. Differences between patients with EMS and pelvic pain and patients with EMS and associated infertility were not statistically significant.

### 3.6. Correlations between CD200 and CD200R Expression on T and B Lymphocyte Subsets and Selected Laboratory Parameters in EMS Patients

**Figure 1:**
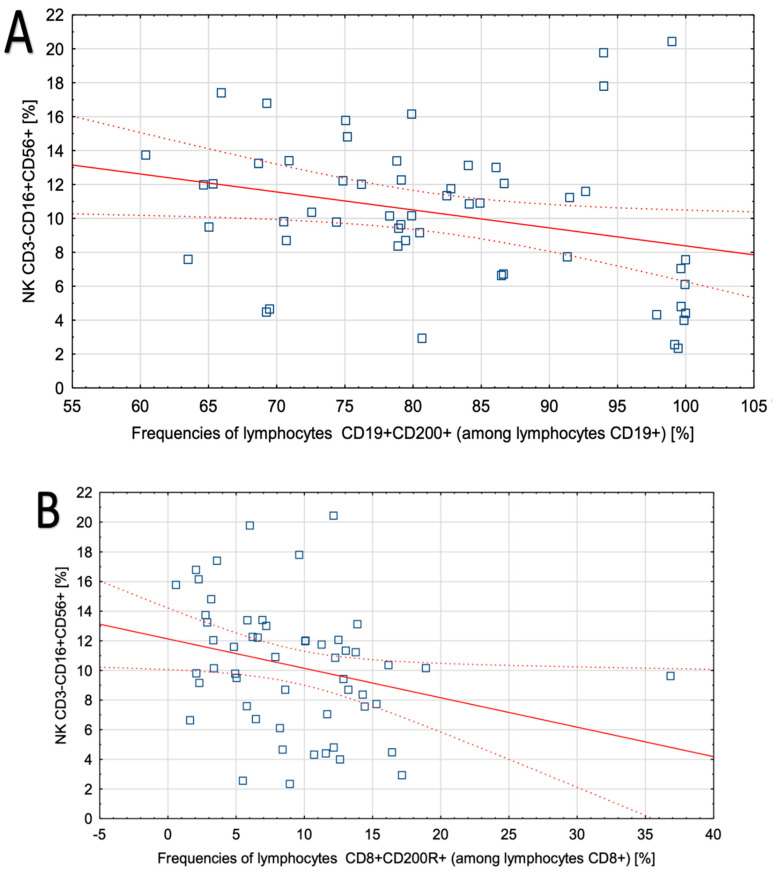
Correlations between the expression of CD200 and CD200R and selected laboratory parameters in patients with the EMS. Spearman correlation coefficients: (**A**) Correlation between the frequencies of CD19+/CD200+ B lymphocytes and CD3+/CD16+/CD56+ NK cells (Pearson’s correlation coefficient; R = −0.33, *p* = 0.013); (**B**) correlation between the frequencies of CD8+/CD200R+ T lymphocytes and CD3+/CD16+/CD56+ NK cells (R = −0.36, *p* = 0.007).

The relationship between CD200 and CD200R expression and laboratory parameters, including white blood cell count, lymphocytes, neutrophils, and monocytes, was analyzed. In patients with EMS, the frequencies of CD19+CD200+ cells and CD8+CD200R+ cells correlated negatively with the concentration of NK cells (Pearson’s correlation coefficient; R= −0.33, *p* = 0.01 and R = −0.36, *p* = 0.007, respectively; Figure 1). The remaining correlations were not significant.

### 3.7. Correlations between CD200 and CD200R Expression on T and B Lymphocyte Subsets in EMS Patients and the Stage of the Disease

The EMS patients were divided into three groups depending on the rASRM stage of the disease, as shown in Table 1. A positive correlation was observed between the disease stage and the percentage of CD8+/CD200+ T lymphocytes (R = 0.522; *p* < 0.001). Additionally, the severity of the disease was weakly correlated with the percentage of CD19+/CD200+ B lymphocytes (R = 0.273; *p* < 0.045). We also found a positive correlation between the degree of disease progression and the percentage of CD8+/CD200R+ T cells (R = 0.400; *p* = 0.003). Table 6 shows all Spearman correlation coefficients.

### 3.8. Univariate and Multivariate Analyses for the Identification of Risk Factors Associated with EMS

We also performed univariate and multivariate analyses to identify risk factors associated with EMS occurrence. In the univariate analysis, CA-125 concentration and the percentage of all analyzed lymphocytes were risk factors for EMS. However, in the multivariate analysis, only CA-125 concentration and the percentage of CD4+CD200+ T lymphocytes were significantly associated with EMS. The results are presented in Table 7.

### 3.9. Receiver Operating Characteristic (ROC) Curve Comparing the Sensitivity and Specificity of CD200 and CD200R in EMS Patients

**Figure 2:**
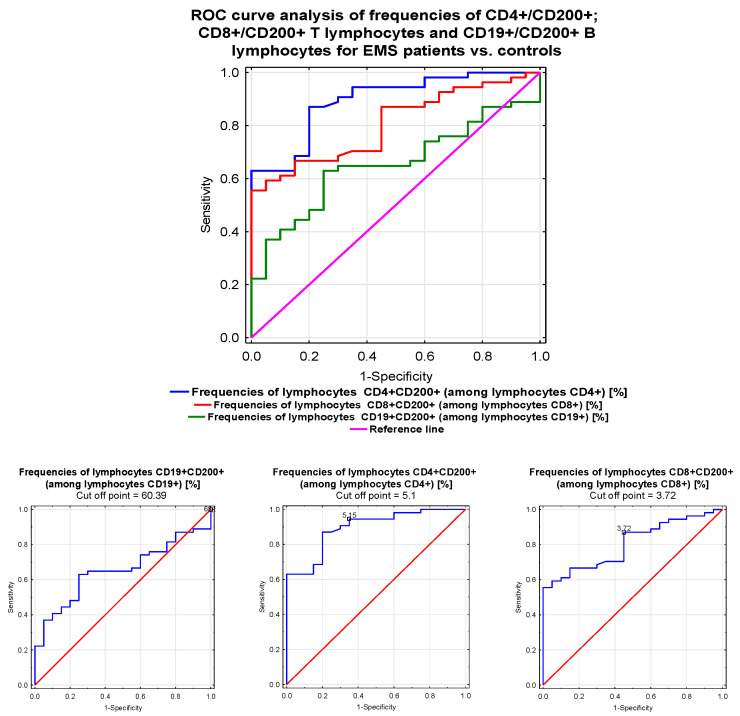
Receiver operating characteristic (ROC) curve analysis showing the sensitivity and specificity of CD200 expression in EMS patients for CD4+/CD8+/CD200+ T lymphocyte frequencies (%) and CD19+/CD200 B lymphocyte frequencies (%).

**Figure 3:**
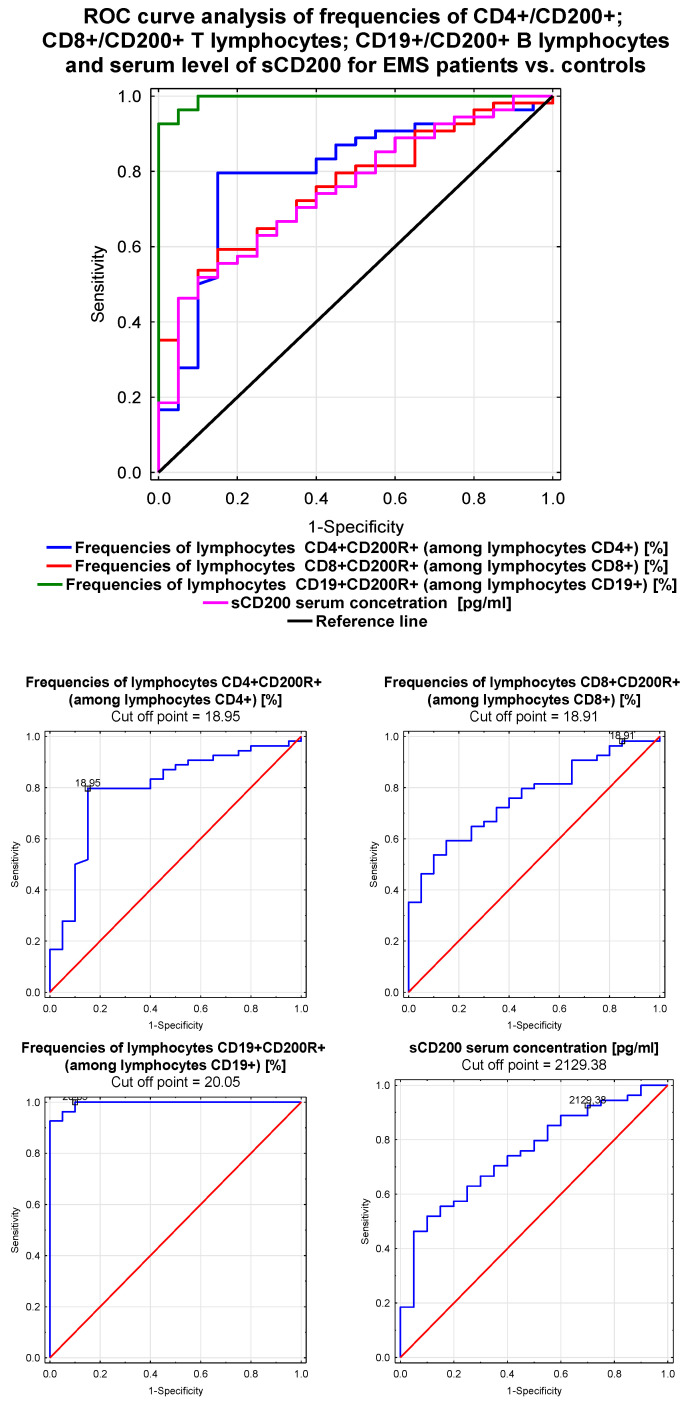
Receiver operating characteristic (ROC) curve analysis showing the sensitivity and specificity of CD200R expression in EMS patients for CD4+/CD8+/CD200R+ T lymphocyte frequencies (%) and CD19+/CD200R lymphocyte frequencies (%).

Figure 2 and Figure 3, as well as Table 8, show the ROC analysis for the immunological parameters related to the CD200 and CD200R molecules. The frequency of CD19+/CD200R B lymphocytes was the most sensitive and specific parameter to identify patients with EMS (AUC = 0.994). The diagnostic accuracy was excellent for increasing frequency of CD4+/CD200 T lymphocytes. Parameters related to CD200R on CD19+ B lymphocytes showed excellent diagnostic accuracy below their prognostic value for the discrimination between patients with EMS and those without.

## 4. Discussion

The impact of the CD200/CD200R-signaling pathway on several inflammatory and autoimmune diseases has been described. Reports point to its role in immune tolerance, macrophage inhibition, and the change in cytokine profile from T helper cells (Th) -1 into Th2, the inhibition of T-cell immunity, and the increase in Treg cell number [23,28,29]. Based on a transgene mouse model overexpressing CD200 Gorczynski et al. [30] suggested that the expression of CD200 played a key role in the modulation of the immune response. They observed that the local and metastatic growth of breast tumor cells was increased in transgenic mice. Moreover, tumors developed more rapidly in these animals and they had a higher metastatic potential than wild-type animals. CD200tg (CD200 transgene) mice were also used as a model for a tissue allograft study. The overexpression of CD200 led to decreased graft rejection. At the same time, it was not necessary to maintain suppression of the established grafts [31,32]. The role of CD200/C200R as a suppressive signaling pathway was also confirmed by Broderick et al. [33], who demonstrated that blocking CD200 was associated with early onset of experimental autoimmune uveitis in mice. Pallasch et al. [34] also found that blocking the CD200-CD200R interaction with anti-CD200 antibodies decreased the population of regulatory T cells. All these data imply that the dysregulation of the CD200/CD200R axis may affect autoimmunity and inflammation.

Inherent to the typical clinical picture of the disease, EMS patients also presented a significantly higher CA-125 concentration. However, due to its low specificity, Ca-125 has limited utility as a marker for EMS [35]. High CA-125 serum concentration may be a sign of epithelial ovarian cancer, especially in combination with existing ovarian lesions [36]. We also demonstrated a significantly higher white blood cell count in the study group compared with the control group, but both results were within the normal range. Interestingly, the assessment of nonspecific immunity parameters showed significantly increased counts of monocytes and neutrophils and a lower percentage of NK cells, indicating an impaired immune response. Neutrophils and macrophages are among the first immune cells to be recruited to the microenvironment of EMS. The physiological response of these cells is probably impaired, though they are the main contributors of elevated proinflammatory cytokines found in the PB or the PF. Kim S.K. et al. [37] reported increased neutrophil count in EMS patients, confirming the inflammatory nature of the disease. Our results are consistent with those reported by others, revealing a decreased number of NK cells in the PB and PF of EMS patients, which may be associated with the reduced cytotoxic activity of these cells [38,39,40]. Interestingly, we also found statistically significant negative correlations between the percentage of NK cells and the frequency of CD8+/CD200+ T lymphocytes and the frequency of CD19+/CD200R+ B lymphocytes, indicating that negative co-stimulation enhances the inhibition of cytotoxicity. A similar observation was made by Coles et al. [26], who reported that CD200 inhibited the function of NK cells in acute myeloid leukemia (AML). Patients with a high expression of CD200 displayed a 50% reduction in NK cell activation compared with patients with a low expression of CD200. Additionally, the antitumor response was enhanced after the use of a CD200-neutralizing antibody in those patients. Ścieżyńska et al. [41] indicated that NK cells may be an immunotherapy target by blocking its negative control checkpoints in EMS patients.

Our study revealed a significantly higher percentage of CD4+/CD200+ T lymphocytes, CD8+/CD200+ T lymphocytes, and CD19+/CD200+ B cells in the study group. At the same time, CD200R antigen expression on the surface of the same lymphocyte populations was significantly lower compared with the control group. The CD200/CD200R-signaling pathway was found to be an important inhibitory mechanism. However, its role as a controller of inflammatory activity in EMS seems to be reduced, because the inhibitory signal of CD200 was transduced by activating the intracellular-signaling motifs of its receptor. Deficient expression of CD200R blocks the effects of the upregulated ligand, resulting in the failure of T cell polarization into a Treg subpopulation and the development of Th17, both involved in the pathogenesis of EMS [17,18,42]. Similar findings were reported by Elshal et al. [43] for Th cells in inflammatory bowel disease (IBD) in pediatric patients. Their study group had decreased CD4+/CD200R1+ T lymphocytes, whereas CD4+/CD200+ T lymphocytes were significantly higher in those patients. Consistent data were presented by other researchers on other systemic autoimmune diseases, including systemic lupus erythematosus (SLE) and rheumatoid arthritis (RA). These data confirm that the altered CD200/CD200R pathway leads to an excessive immune response in both inflammatory and autoimmune disorders [44,45]. EMS has not been classified as an autoimmune disease, but there is high-quality evidence confirming a significant association between EMS and SLE, RA, and IBD [46]. The abovementioned findings were unrelated to disease activity, in contrast to our study, in which positive correlations between the stage of EMS and the percentages of CD8+/CD200+ T lymphocytes, CD19+/CD200+ B lymphocytes, and CD8+/CD200R+ T cells were observed. CD200 is one of the common markers of exhausted T lymphocyte immunophenotype [47]. Lymphocyte-mediated cytotoxicity is crucial for the clearance of ectopic endometrial implants. The progression of EMS is associated with an increasing level of this mechanism dysfunction, which may be related to the chronic inflammation accompanying the disease [48]. Hyporesponsiveness of T CD8+ cells may be contributing to their exhaustion [49]. In our previous research, we showed that advanced EMS was characterized by higher frequencies of programmed death-1 (PD-1)-positive T and B cells, which were the hallmark of T cell exhaustion [50]. Our results are interesting in light of a recently published study, in which CD200 overexpression on AML blasts suppressed CD4+ and CD8+ T lymphocyte function via the CD200/CD200R pathway. Blocking this mechanism only partially restored T cell activity, which suggests the involvement of another immunosuppressive molecule. Further studies revealed that co-expression of both CD200 and programmed death ligand-1 (PD-L1) resulted in a 90% reduction in T cell activation. Our data confirmed for the first time that the CD200/CD200R pathway and the PD-1/PD-L1 axis co-expressed on T cells can multiply the immunosuppressive effect [51].

Due to the complex nature of EMS, the influence of the CD200/C200R-signaling pathway on its development remains poorly understood. Very few available publications describe the role of this pathway in EMS. Weng et al. [52] reported that the plasma concentration of CD200 in patients with EMS was significantly increased. These results differ from ours, since we found a significantly lower concentration of CD200 in the study group compared with the control group. However, their study group consisted of only seven patients, all with ovarian EMS. The small group size and the focus on a single type of lesion could have also influenced other results by Weng et al. Moreover, their results showed that the expression of CD200 was upregulated in ectopic endometrium compared with normal eutopic endometrium. The expression of CD200R on peritoneal macrophages was enhanced in patients with EMS. In that study, CD200 expression in human endometrial stromal cells stimulated with estrogen was higher, while macrophage phagocytosis in vitro decreased. The authors concluded that CD200 was an important immunosuppressive marker in EMS and it might promote the development of the disease via the inhibition of phagocytosis. Clark et al. [53] analyzed CD200 and CD200R1 expression in eutopic and ectopic deposits of EMS and in deposits of adenomyosis, with immunohistochemistry staining. They observed a different staining intensity in ectopic peritoneal implants and cystic forms in the tissue of EMS patients. No statistically significant differences were detected in CD200 and CD200R expression in full-thickness eutopic endometrium of EMS patients and controls in the proliferative and secretory phases. The authors concluded that the CD200 staining intensity was not useful for the identification of patients who are at risk of developing EMS. They also suggested that the level of sCD200 in menstrual blood might be a new marker for diagnosis and treatment. However, the significantly lower sCD200 serum concentration obtained in this study indicates its limited utility as a marker of disease severity in EMS.

Our results show that the expression of CD200 and CD200R on lymphocyte subsets, particularly on CD19+ B lymphocytes (measured by flow cytometry), may be a good marker enabling the differentiation between EMS patients and healthy controls. While several studies have shown expression of CD200/CD200R in ectopic endometrial tissue, a means to distinguish between patients in a less invasive and time-consuming manner is still needed [53,54].

Our observations are promising; however, they require further validation. In particular, repeated within-subject analyses should examine whether the test results are stable. Including a greater number of participants in the study and control groups should also be considered. Because various clinical presentations of endometrial implants may be associated with different pathogeneses, dividing the study group according to the type of EMS should be considered [55,56]. Additionally, the CD200/CD200R pathway should be analyzed in patients not treated postoperatively with medications that may affect the immune system. The assessment of the association between CD200 and CD200R expression and EMS recurrence is also necessary.

## 5. Conclusions

Our study revealed statistically significant differences in the expression of CD200 and CD200R on selected lymphocyte subsets in patients with EMS, which correlated with the stage of the disease. The assessment of PB lymphocytes expressing CD200 and CD200R, as well as of the sCD200 concentration, indicates these molecules play an important role as negative co-stimulators in the induction and the persistence of inflammation associated with EMS. Similar findings in the context of several autoimmune diseases imply that the dysregulation of the CD200/CD200R axis may be involved in their pathogeneses. CD200 and CD200R expression may serve as a marker to distinguish between EMS cases. The usefulness of CD200 and CD200R as markers in the management of EMS should be further investigated.

## Figures and Tables

**Table 1:** Characteristics of the study and the control groups.

Parameter	EMS (*n* = 54)	CONTROL (*n* = 20)	*p*-Value
Age [years] mean ± SD	35.00 ± 6.19	36.65 ± 6.87	NS
Disease stage:		N/A	-
I	31.48% (*n* = 17)		
II	31.48% (*n* = 17)		
III–IV	37.04% (*n* = 20)		
Adhesions	57.41% (*n* = 31)	N/A	-
Dysmenorrhea	79.63% (*n* = 43)	N/A	-
Infertility	50.00% (*n* = 27)	N/A	-
CA-125 [U/mL] mean ± SD	37.79 ± 28.95	9.23 ± 5.37	*p* < 0.005
HE-4 [pmol/L] mean ± SD	39.96 ± 9.69	37.05 ± 9.14	NS
TSH serum concentration [µlU/mL] mean ± SD	1.38 ± 0.63	1.43 ± 0.69	NS
FT3 serum concentration [pg/mL] mean ± SD	3.15 ± 0.57	3.13 ± 0.58	NS
FT4 serum concentration [ng/mL] mean ± SD	1.31 ± 0.24	1.30 ± 0.22	NS
Estradiol serum concentration [pg/mL] mean ± SD	57.93 ± 26.43	56.23 ± 27.43	NS
FSH serum concentration [mlU/mL] mean ± SD	5.37 ± 1.80	6.17 ± 1.58	NS
LH serum concentration [mlU/mL] mean ± SD	6.64 ± 2.76	5.96 ± 1.82	NS
BMI [kg/m^2^] mean ± SD	21.8 ± 2.12	21.87 ± 1.84	NS

NS—not significant; N/A—not applicable; EMS—endometriosis; CA-125—cancer antigen 125; HE-4—human epididymis protein 4.

**Table 2:** White blood cell count and basic lymphocyte subsets in patients with endometriosis (EMS) and controls.

Parameter	EMS (*n* = 54)		CONTROL (*n* = 20)		*p*-Value
	Mean ± SD	Median (Range)	Mean ± SD	Median (Range)	
WBC (10^3^/mm^3^) (normal range: 4.0–10.0)	8.36 ± 1.58	8.26 (5.51–11.33)	7.42 ± 0.77	7.31 (6.37–8.66)	0.015
LYM (10^3^/mm^3^) (normal range: 1.12–4.73)	2.24 ± 0.73	2.06 (1.20–3.83)	2.44 ± 0.45	2.54 (1.53–3.07)	NS
NEU (10^3^/mm^3^) (normal range: 1.12–4.73)	5.04 ± 1.40	5.50 (2.08–7.91)	4.32 ± 1.03	3.94 (2.71–6.03)	0.015
MON (10^3^/mm^3^) (normal range: 1.12–4.73)	0.54 ± 0.17	0.56 (0.24–0.86)	0.47 ± 0.09	0.49 (0.28–0.59)	0.054
CD3+ T lymphocytes (%)	70.83 ± 4.63	71.86 (61.31–78.77)	68.26 ± 3.84	68.08 (60.63–74.49)	0.021
CD19+ B lymphocytes (%)	10.55 ± 3.11	9.76 (6.12–16.84)	11.25 ± 2.50	11.40 (6.04–16.90)	NS
CD3+/CD4+ T lymphocytes (%)	43.09 ± 7.64	44.01 (26.13–65.45)	44.46 ± 2.51	44.16 (40.71–48.84)	NS
CD3+/CD8+ T lymphocytes (%)	28.07 ± 6.85	27.99 (16.25–42.90)	34.36 ± 3.29	34.74 (29.33–39.60)	<0.005
T CD3+/CD4+: T CD3+/CD8+ lymphocyte ratio	1.67 ± 0.65	1.59 (0.67–3.88)	1.31 ± 0.16	1.29 (1.03–1.57)	0.016
T regulatory cells CD4+/CD25+/FOXP3 (%)	6.30 ± 3.16	5.46 (0.39–13.55)	6.20 ± 2.02	6.25 (3.13–9.68)	NS
NK cells CD3-CD16+CD56+ (%)	10.31 ± 4.30	10.26 (2.34–20.43)	15.35 ± 2.25	14.43 (12.16–19.34)	<0.001
NKT-like cells CD3+/CD16+/CD56+ (%)	3.40 ± 2.85	2.27 (0.21–11.26)	3.02 ± 1.02	3.27 (1.15–4.92)	NS

EMS—endometriosis; WBC—white blood cell count; LYM—lymphocytes; NEU—neutrophils; MON—monocytes; NK—natural killer.

**Table 3:** The sCD200 serum concentration and frequency of CD200 and CD200R expression on T and B lymphocytes in EMS patients and controls.

Parameter	EMS (*n* = 54)		CONTROL (*n* = 20)		*p*-Value
	Mean ± SD	Median (Range)	Mean ± SD	Median (Range)	
CD19+CD200+ B lymphocytes (%)	81.81 ± 11.58	79.90 (60.39–99.99)	76.16 ± 6.55	76.59 (67.39–93.19)	0.04
CD4+CD200+ T lymphocytes (%)	10.55 ± 3.11	9.76 (6.12–16.84)	11.25 ± 2.50	11.40 (6.04–16.90)	<0.001
CD8+CD200+ T lymphocytes (%)	9.42 ± 6.23	7.40 (1.88–28.04)	3.88 ± 1.56	3.66 (0.35–6.45)	<0.001
CD19+CD200R+ B lymphocytes (%)	10.92 ± 3.82	11.05 (4.15–20.05)	25.02 ± 4.06	24.80 (16.91–31.89)	<0.001
CD4+CD200R+ T lymphocytes (%)	14.60 ± 7.87	14.44 (3.23–40.71)	23.12 ± 7.95	22.28 (7.39–38.83)	<0.001
CD8+CD200R+ T lymphocytes (%)	9.19 ± 6.07	8.51 (0.59–36.84)	14.38 ± 5.78	13.24 (6.03–31.92)	<0.001
sCD200 serum level (pg/mL)	964.85 ± 682.92	742.05 (63.82–3037.54)	1774.53 ± 1185.13	1339.94 (513.81–4784.76)	0.001

EMS—endometriosis; CD200- cluster of differentiation-200; CD200R- receptor of cluster of differentiation-200; sCD200—soluble cluster of differentiation-200.

**Table 4:** The sCD200 concentration and frequencies of CD200 and CD200R on T and B lymphocytes in patients with different types of EMS: Endometrioma, deep infiltrating EMS, and peritoneal EMS.

Parameter	Endometrioma (Mean ± SD/Median; Range)			DIE (Mean ± SD/Median; Range)			Peritoneal Endometriosis (Mean ± SD/Median; Range)		
	Positive (*n* = 30)	Negative (*n* = 24)	*p*-Value	Positive (*n* = 11)	Negative (*n* = 43)	*p*-Value	Positive (*n* = 35)	Negative (*n* = 19)	*p*- Value
CD19+CD200+ B lymphocytes (%)	79.24 ± 12.36/ 78.6; 60.4–99.99	85.01 ± 9.86/ 82.6; 66.7–99.99	0.049	92.46 ± 12.06/ 99.2; 69.2–99.99	79.08 ± 9.9/79.1; 60.4–99.99	0.00034	84.3 ± 10.3/82.8; 64.6–99.99	77.3 ± 12.7/ 74.9; 60.4–99.99	0.029
CD4+CD200+ T lymphocytes (%)	11.37 ± 5.25/ 10.5; 3.5–24.3	12.95 ± 7.2/ 11.3; 4.3–34.7	0.53	15.02 ± 8.77/ 13.6; 3.5–34.7	11.32 ± 5.2/ 9.98; 4.3–26.1	0.076	11.8 ± 6.3/ 9.4; 3.5–34.7	12.6 ± 5.3/ 12.4; 4.6–24.3	0.33
CD8+CD200+ T lymphocytes (%)	9.48 ± 5.7/ 7.4; 2.3–25.1	9.36 ± 6.97/ 7.6; 1.2–28.0	0.53	15.24 ± 7.54/ 16.3; 2.3–28.0	7.94 ± 4.9/ 6.3; 1.9–25.1	0.00027	8.5 ± 6.3/ 6.6; 1.9–28.0	11.1 ± 5.9/ 12.2; 3.3–25.1	0.057
CD19+CD200R+ B lymphocytes (%)	9.98 ± 3.56/ 10.0; 4.2–20.1	12.1 ± 3.9/ 11.7; 5.3–19.2	0.05	11.66 ± 3.67/ 11.6; 6.7–17.8	10.73 ± 3.9/ 10.8; 4.2–20.1	0.48	11.4 ± 3.8/ 11.2; 5.3–20.1	10.0 ± 3.9/ 10.4; 4.2–17.8	0.25
CD4+CD200R+ T lymphocytes (%)	15.36 ± 9.02/ 14.5; 3.2–40.7	13.66 ± 6.19/ 13.9; 3.9–23.8	0.18	13.3 ± 5.36/ 14.4; 4.3–20.7	14.94 ± 8.4/ 14.5; 3.2–40.7	0.54	13.9 ± 6.4/ 14.4; 3.2–29.9	15.9 ± 10.1/ 14.5; 4.3–40.7	0.77
CD8+CD200R+ T lymphocytes (%)	9.24 ± 7.26/ 7.6; 0.6–36.8	9.12 ± 4.3/ 9.3; 2.3–17.2	0.67	12.28 ± 3.67/ 12.1; 5.5–17.2	8.40 ± 6.4/ 6.9; 0.6–36.8	0.058	9.4 ± 6.3/ 8.6; 1.6–36.8	8.8 ± 5.7/ 6.9; 0.6–18.9	0.94
sCD200 serum level (pg/mL)	1091.21 ± 689.1/ 881.9; 63.8–3037.5	806.9 ± 655.1/5 48.8; 125.6–2667.9	0.021	1029.63 ± 850.0/ 611.0; 506.7–3037.5	948.28 ± 644.3/ 816.3; 63.8–2960.3	0.76	903.6 ± 612.5/ 751.0; 125.6–2667.9	1077.7 ± 802.4/ 733.1; 63.8–3037.5	0.49

EMS—endometriosis; DIE—deep infiltrating endometriosis.

**Table 5:** The sCD200 serum concentration and frequencies of CD200 and CD200R on T and B lymphocytes in patients with EMS with concomitant adhesions, pelvic pain, or infertility.

Parameter	Adhesions (Mean ± SD/Median; Range)			Pelvic pain (Mean ± SD/Median; Range)			Infertility (mean ± SD/Median; Range)		
	Positive (*n* = 31)	Negative (*n* = 23)	*p*-Value	Positive (*n* = 43)	Negative (*n* = 11)	*p*-Value	Positive (*n* = 27)	Negative (*n* = 27)	*p*-Value
CD19+CD200+ B lymphocytes (%)	85.3 ± 11.7/ 80.7; 65.9–99.99	77.1 ± 9.9/ 79.1; 60.4–92.7	0.024	83.0 ± 12.2/ 79.9; 63.5–99.99	77.3 ± 7.8/ 79.9; 60.4–88.6	0.27	80.3 ± 10.2/ 79.9; 64.2–99.9	83.3 ± 12.8/ 79.5; 60.4–99.99	0.47
CD4+CD200+ T lymphocytes (%)	13.6 ± 7.2/ 12.4; 3.5–34.7	10.0 ± 3.8/ 9.1; 4.3–18.9	0.077	12.8 ± 6.6/ 11.1; 3.5–34.7	9.4 ± 2.8/ 8.8; 4.6–13.5	0.11	12.4 ± 6.2/ 10.1; 5.2–34.7	11.7 ± 6.2/ 10.8; 3.5–26.1	0.56
CD8+CD200+ T lymphocytes (%)	11.5 ± 6.8/ 9.8; 2.3–28.0	6.7 ± 4.0/ 5.3; 1.9–15.0	0.0056	10.3 ± 6.5/ 8.3; 1.9–28.0	6.1 ± 3.6/ 4.5; 2.9–14.1	0.057	9.0 ± 5.6/ 7.1; 2.9–14.0	9.8 ± 6.8/ 7.5; 1.9–28.0	0.84
CD19+CD200R+ B lymphocytes (%)	10.1 ± 3.8/ 9.1; 4.2–20.1	12.0 ± 3.7/ 12.2; 5.4–19.2	0.059	10.6 ± 3.8/ 10.5; 4.2–20.1	12.2 ± 3.7/ 11.2; 6.0–19.2	0.22	11.2 ± 3.9/ 11.2; 5.2–19.2	10.7 ± 3.8/ 11.0; 4.2–20.1	0.63
CD4+CD200R+ T lymphocytes (%)	15.4 ± 8.4/ 15.0; 3.2–40.7	13.5 ± 7.2/ 12.7; 3.9–29.9	0.38	15.0 ± 8.3/ 14.8; 3.2–40.7	12.9 ± 5.7/ 12.7; 5.2–22.7	0.48	13.8 ± 6.9/ 13.5; 3.9–29.9	15.4 ± 8.8/ 14.5; 3.2–40.7	0.74
CD8+CD200R+ T lymphocytes (%)	10.7 ± 6.8/ 10.7; 0.6–36.8	7.1 ± 4.3/ 6.2; 2.1–15.3	0.024	9.9 ± 6.2/ 8.9; 0.6–36.8	6.6 ± 4.8/ 4.9; 1.2–13.0	0.071	9.4 ± 5.2/ 10.1; 2.1–18.9	8.9 ± 6.9/ 8.2; 0.6–36.8	0.43
sCD200 serum level (pg/mL)	972.1 ± 733.4/ 732.5; 63.8–3037.5	955.1 ± 624.4/ 889.0; 125.6–2667.9	0.81	1035.1 ± 731.9/ 751.0; 63.8–3037.5	690.0 ± 342.5/ 649.0; 140.2–1214.0	0.28	1019.9 ± 772.8/ 816.3; 125.6–3037.5	909.8 ± 589.2/ 733.1; 63.8–2960.3	0.89

EMS—endometriosis.

**Table 6:** Correlations between the stage of the disease and lymphocytes expressing CD200 or CD200R.

Correlation between the Stage of Ems and Lymphocytes Expressing CD200 or CD200R	*R*	*p*-Value
CD19 + CD200+ B lymphocytes (%)	0.273	0.045
CD4 + CD200 + T lymphocytes (%)	0.183	0.185
CD8 + CD200 + T lymphocytes (%)	0.522	<0.001
CD19 + CD200R + B lymphocytes (%)	−0.076	0.583
CD4 + CD200R + T lymphocytes (%)	0.015	0.913
CD8 + CD200R + T lymphocytes (%)	0.400	0.003

EMS—endometriosis.

**Table 7:** Results from the univariate and multivariate analyses for the identification of risk factors associated with EMS.

Parameter	Univariate Analysis		Multivariate Analysis	
	OR (95% CI)	*p* Value	OR (95% CI)	*p* Value
Age [years]	0.96 (0.89–1.0)	0.32		
BMI (kg/m^2^)	0.98 (0.76–1.3)	0.89		
CA-125 concentration [U/mL]	1.31 (1.1–1.5)	0.0004	1.42 (1.1–1.8)	0.004
HE4 concentration [pmol/L]	1.04 (0.98–1.1)	0.25		
CD19+CD200+ B lymphocytes (%)	1.06 (1.0–1.12)	0.0496		
CD4+CD200+ T lymphocytes (%)	1.78 (1.3–2.4)	0.0001	1.71 (1.2–6.1)	0.016
CD8+CD200+ T lymphocytes (%)	1.58 (1.2–2.1)	0.0035		
CD19+CD200R+ B lymphocytes (%)	0.39 (0.20–0.76)	0.0059		
CD4+CD200R+ T lymphocytes (%)	0.88 (0.82–0.95)	0.0009		
CD8+CD200R+ T lymphocytes (%)	0.87 (0.78–0.96)	0.0077		
sCD200 serum level (pg/mL)	0.99 (0.998–1.0)	0.0034		

**Table 8:** Receiver operating characteristic (ROC) analysis to determine the diagnostic accuracy of immunological parameters in EMS patients.

Parameter	AUC	95% CI	PPV	NPV	LR(+)	LR(−)	ACC	*p*-Value *
CD4+/CD200 + lymphocytes [%]	0.895	0.82–0.969	92.7	54.5	3.09	0.42	0.656	*p* < 0.0001
CD8+/CD200 + lymphocytes [%]	0.804	0.706–0.903	90.3	45.1	2.80	0.51	0.618	*p* < 0.0001
CD19+/CD200 + lymphocytes [%]	0.653	0.528–0.778	84.3	30.5	2.29	0.77	0.566	*p* = 0.0165
CD4+/CD200R + lymphocytes [%]	0.800	0.684–0.917	88.4	44.6	3.11	0.52	0.622	*p* < 0.0001
CD8+/CD200R + lymphocytes [%]	0.761	0.651–0.872	88.3	41.8	2.92	0.55	0.606	*p* < 0.0001
CD19+/CD200R + lymphocytes [%]	0.994	0.984–1.0	95.6	62.0	5.34	0.36	0.698	*p* < 0.0001
sCD200 concentration [pg/mL]	0.750	0.632–0.868	87.3	43.3	3.16	0.55	0.602	*p* < 0.0001

* DeLong test for comparison with no effect (AUC = 0.500). ROC—receiver operating characteristic; EMS—endometriosis; AUC—area under the curve; CI—confidence interval; PPV—positive predictive value (%); NPV—negative predictive value (%); LR—likelihood ratio; ACC—accuracy

## Abbreviations

AML	acute myeloid leukemia
BMI	body mass index
Ca-125	cancer antigen 125
CD4+	cluster of differentiation 4+
CD200	cluster of differentiation-200
CD200R	receptor of cluster of differentiation-200
CD200tg	CD200 transgene
DCs	dendritic cells
EDTA	ethylenediaminetetraacetic acid
EMS	endometriosis
HE-4	
IDO	indoleamine-pyrrole 2,3-dioxygenase
Ig	immunoglobulin
Ig SF	immunoglobulin superfamily
kDA	kilodaltons
LYM	lymphocytes
MON	monocytes
N/A	not applicable
NEU	neutrophils
NS	not significant
NK	natural killer cells
PB	peripheral blood
PD-1	programmed death-1
PD-L1	programmed death ligand-1
PF	peritoneal fluid
ROC	receiver operating characteristic
sCD200	soluble CD200
Th	T helper cells
Tregs	T regulatory cells
WBC	white blood cells
